# Hammering with the telescope

**DOI:** 10.3389/frai.2022.1010219

**Published:** 2022-11-25

**Authors:** Pawel Sobkowicz

**Affiliations:** NOMATEN Centre of Excellence, National Centre for Nuclear Research, Otwock, Poland

**Keywords:** Artificial Intelligence, machine learning, law, crime, ethics

## Abstract

The rapid pace in which various Artificial Intelligence and Machine Learning tools are developed, both within the research community and outside of it, often discourages the involved researchers from taking time to consider potential consequences and applications of the technical advances, especially the unintended ones. While there are notable exceptions to this “gold rush” tendency, individuals and groups providing careful analyses and recommendations for future actions, their adoption remains, at best, limited. This essay presents an analysis of the ethical (and not only) challenges connected with the applications of AI/ML methods in the socio-legal domain.

## 1. Introduction

### 1.1. Personal foreword

This essay is the result of an almost knee-jerk reaction to the beautiful metaphor used as the topic of this special issue. The telescope is a wonderful scientific invention. Galileo used it to discover the moons of Jupiter and the “appendages” of Saturn, revolutionizing astronomy. And this is what most of us remember from history lessons. But we should recall that in his first letter to Doge of Venice, Galileo pointed out a very different use: “*this telescope has the advantage of discovering the ships of the enemy 2 h before they can be seen with the natural vision and to distinguish the number and quality of the ships and to judge their strength and be ready to chase them, to fight them, or to flee from them*” (Galilei, 1609).

Of course, sufficiently heavy and robust brass classical telescope may be used quite effectively as a “blunt instrument” to harm someone. The shape is just right. But much more importantly, it can be used more “artfully” to create even greater harm in the military campaigns. And, as we know, such “naval” use of the telescopes exceeded the scientific ones.

Just like the telescope, Artificial Intelligence (AI) tools and discoveries may have their dual use. In this essay, I will attempt to gather thoughts about the balance between good and evil uses of AI in the socio-legal context^1^ and the potential consequences for the research community.

### 1.2. Ubiquity of AI: Breakthrough, useful, fashionable, or dangerous?

In a series of technological advancements, modern societies have transitioned to a situation unprecedented in history. The major part of this revolution relies on the transfer of many (most?) human activities to the Internet domain. Our digital trail provides accessible data that can be used to document, analyse, and even predict our behaviors. Big Data analyses (using real-world, human relevant data), When combined with ML/AI tools advances, can to go much further than before (Yarkoni and Westfall, 2017; Chen et al., 2021). The social and ethical impact of the AI/ML development has naturally been realized before (for example in the works of the multinational team which has created the Montréal Declaration for Responsible AI Abrassart et al., 2018; Dilhac et al., 2018), but despite these efforts much of the research devoted to creation of new tools and methods treats the nontechnical aspects as secondary. Moreover, the advances driving these changes are no longer only in the hands of the research community. Many significant developments are achieved by commercial companies and governmental institutions. The ethical restrictions created for scientists and research institutions are absent for governments and commercial companies.

Moreover, there is a tremendous gap in data accessibility between commercial companies hosting social network data (e.g., Meta/Facebook, Twitter, Google) and the research community (not to mention the differences in available funds). The disparity touches thus both the algorithms and data. We are obliged to be transparent, provide open access and remain within specific ethical boundaries; they (the companies and governments) are not.

Driven partially by this digital revolution and in part by conceptual and technical advances, AI has invaded practically all domains of research, development and innovation activities in the past decade. From cosmology (Carleo et al., 2019) to sociology (Mützel, 2015; McFarland et al., 2016; Molina and Garip, 2019), it is hard to find a topic in which Machine Learning (ML) and Artificial Intelligence have not been used (or at least promised to be used). In some domains, the benefits and gains achieved by AI are tangible and clear: feats which were impossible or prohibitively costly are now commonplace and fast. In other fields, one could still state that invoking AI in grant applications or publications is a matter of going with the fashion trends, and the benefits are (yet?) illusory. The latter situation is, for obvious reasons, rarely admitted. A similar reluctance covers the dangers resulting from the use of AI tools, but fortunately, this is increasingly recognized.

As a result, the relationship between scientific understanding of phenomena related to “digital humanity” and the practical (commercial or governmental) uses of such knowledge and the associated tools and algorithms is asymmetrical. The tools created by scientists (*the telescopes*) can be indiscriminately used by other, less conscientious players (becoming *the hammers* needed to shape some desired societal outcomes).

This phenomenon has been noted in the context of sociophysics (Agent Based Models of social behaviors) (Sobkowicz, 2019), and it also applies to AI in legal and socio-legal domains. There is significant asymmetry of accessibility of data and access to advanced AI tools present between citizens or small businesses on one side and the governments or large companies (in particular the social communication behemoths). It can make the principle of equality under the law impossible to maintain. This phenomenon is already seen in some countries (one can mention the Chinese “social credit” system). When some actors have almost unlimited access to the personal data of others, the temptation to use it is hard to resist.

The research community is thus faced with a moral choice: to continue the research, including the creation of new analytical tools, algorithms, and predictive models or to pause the research, until humanity becomes ready for it. There are examples of the second choice already in place (e.g., in research of cloning, embryo manipulation, etc.)—but in all cases, there were detractors who continued the banned research for various reasons.

There is also a third choice, much more challenging and difficult to implement: to focus the attention on the development of *methods and tools that would by design act in the reverse direction*, strengthening the position of individual people against governments, corporations, and large institutions. Just as the legal equality principle promotes the right to professional legal representation, we could think of designing systems that could counterbalance the present asymmetry by being available and usable by everyone.

### 1.3. AI/ML research: Development vs. usage stage

To focus our attention, let's clearly distinguish the stages associated with AI. The first is the *creation* of the tools, subsystems, libraries, training datasets, and convergence/optimization processes. The research community plays an active role at this stage. It's Galileo polishing the lenses, improving the tubes, to create better and better telescopes. There are extremely interesting research challenges specifically related to socio-legal domain (such as using AI when data are inherently incomplete or contradictory, see, e.g., Section 2.1). The key actors at this stage are computer scientists, but as the experience and knowledge of AI/ML in other research disciplines grows, other researchers may play an important role as well. Especially with respect to system using specific data (audio-visual, natural language, medical etc.). As noted above, the research is not limited to academia—the efforts of commercial and governmental institutions (e.g., military) are leading in some domains, thanks to both better data access and funding.

The second stage is the practical *use* of AI tools—which can still be research oriented (Galileo studying planets and constellations) or practical (telescopes in marine service). In many research funding programs it is *expected* that the results of academic research would be useful and beneficial to the society as a whole and/or turned into products and solutions with economic impact. Grant applications get points for such an “impact” score. And it is relatively easy to promise some commercial potential of AI in almost any social environment. In my personal experience of reviewing over a thousand grant proposals in the past few years, about one third included AI/ML component. In many cases the research plan could do quite well without it, or the innovative contribution to/from AI was minimal—but still the proposal Authors deemed it crucial to include AI as a necessary ingredient in potential proposal's success.

Even for successful funding applications, the promise of commercialization and economic impact is not always fulfilled. But, quite often, the outcomes of the academic research, for example algorithm improvements, dedicated libraries or datasets usable for training AI are openly published with liberal licenses. They can be used by third parties, sometimes in applications very different from what the creators had in mind. Once distributed, they are quite hard to monitor and control.

Is then the focus on AI in research a trendy fad, or a useful and deep change in the available research instrumentation, leading to a fundamental transformation of how science is done and used? Science has had similar fashions before in different disciplines, but probably none could compare with the breadth of the AI/ML presence. It is rather obvious that the contribution will grow in importance in some fields, but for the others, the future is less certain. Not only due to the doubts about improvements brought into the research practice by AI/ML, but also because the potential applications in social and economic domains are questionable from practical, psychological, and ethical points of view.

### 1.4. AI in socio-legal context: The basic usage scenarios

The applications of AI in the “legal-related” fields fall into the “difficult' category mentioned above. Partially it is because societal life is extremely complex to study and understand, but even more importantly, because we are used to reserving these domains for human deliberations and decisions. Still, in the past decades, data-driven analyses and then ML/AI tools were introduced in a number of contexts (Surden, 2014, 2019; Stern, 2018; Rigano, 2019; Sil et al., 2019). Some examples are listed below.

- Support for day-to-day activities in legal professions: data search, legal knowledge analyses, case pre-evaluation, etc.
- Decision making (or, more often, support of human decision making) by legal administration or judges (e.g., in bail, sentencing or probation cases).
- Decision making (often autonomous and only formally supervised by humans) in economic decisions (e.g., credit scoring).
- Analysis of data in both court and economic environments, including the search for potential case argumentation, is important especially for processing large amounts of digital data (search for discrepancies, proofs of specific behaviors etc.).
- Similar analysis in the context of informal processes, such as social network data^2^—with important social outcomes (sifting through personal or organizational digital trails to dig information needed to attack a given person or community on social media and beyond).
- Police (and related forces) profiling of individuals and communities—either during ongoing investigations or in so-called predictive policing.
- Use of AI/ML algorithms (such as facial recognition or phone/network monitoring) in monitoring general population activities by authoritarian regimes.
- Data manipulation (especially online), creation of deep-fakes and other false “evidence” aimed at various goals, from politics through fraud and crime to personal quarrels and vendettas.

The fact that AI/ML tools are often black-box models makes the situation even worse: whether the systems take “final” decisions or only provide an “objective background” for human actors, the influence of the technology may determine the lives of individuals or groups. This has led to the calls for increased ethical scrutiny of the applications (Dreyling et al., 2021), or for building explainable models (Bibal et al., 2021).

The need for Explainable AI (xAI), designed in a way that would allow humans to understand how the algorithm reaches its conclusions and to predict its behavior, has been increasingly recognized. The European Commission (EC2, 2019) included the following requirements: “*the traceability of AI systems should be ensured; it is important to log and document both the decisions made by the systems, as well as the entire process (including a description of data gathering and labeling, and a description of the algorithm used) that yielded the decisions. Linked to this, explainability of the algorithmic decision-making process, adapted to the persons involved, should be provided to the extent possible*.” An in-depth analysis of the potential dangers is also provided by the EC White Paper on AI (EC2, 2020). US government agencies take equally serious view of the need for explainability and transparency, coupling them with the needs for AI systems verification, validation, security under potential attacks, alignment of values and long-term safety (NAT, 2019).

Can explainability prevent or minimize ethical risks of using AI in social contexts? The answer is not trivial, and any effects rely on the level of trust that the society at large puts in the researchers. Because what is explainable to specialists is not necessarily so to the general public. So, most likely, while the ability to understand how an AI reaches its conclusions is a necessary condition to monitor all aspects of its use, it is not a sufficient condition.

### 1.5. What's so special about law?

Ubiquity of AI applications is already a fact. Quite often we (as citizens) do not even notice its presence and effects. Or perceive AI as some marketing slogan, as in our mobile phone cameras and other gadgets. Why then should we treat the legal domain any different from engineering, entertainment, banking or health services?

Law has a special role in social life. It combines the communal (even global) scale and individual cases and impacts. The latter may touch deeply personal matters, transforming abstract (and often incomprehensible) language and processes into emotional, life-defining experiences. Democratic societies have gradually developed tools aimed at ensuring “equality under the law.” These include processes and institutions devoted to equality in access/privileges, due process provisions and protection from abuse.

The importance of the legal institutions and processes may be compared to the health domain. Like in medicine, the complexity of the modern legal systems puts all of us at the “mercy” of specialists and experts. Some of the measures mentioned above aim to ensure that access to such expert advice is universal, but let's not fool ourselves: the resources and expertise follow power and money. Rich people can afford better medical care and better lawyers, at least in some cases.

One can thus ask if introducing another level of complexity, provided by mysterious (for most of us) workings of AI will be universally beneficial, or will it favor the rich and powerful? Do we want the AI to plan a significant role in decisions related to the activities listed above? Will we trust such decisions (an important question in the context of lack of trust in human lawyers).

With respect to the research community, these doubts may translate to consideration of how we should develop the ML tools and applications related to legal systems in our countries. Or even more: this essay is written from the perspective of a democratic country. But scientific discoveries and developments know no boundaries. Telescopes were used by pirates as well. Should we take into account how our achievements and improvements could be used by autocracies, dictators, terrorists?

## 2. “People lie.” Deal with it, AI

An interesting case is presented by the potential use of AI in civil and criminal proceedings. Not just the analyses of the accumulated legal documents (important especially in the US precedent based legal system), but applied directly to the evidence in specific trials.

And here we come to the stage where for centuries or more, human intelligence has not been generally good enough. The judiciary system deals with the conflict of opposing views. The institutions of the mandatory access to legal counsel and the right to appeal reflect the simple statement that decisions are (in many cases) far from obvious. The information available to judges and jurors is muddled, incomplete, and often contradictory, or inconclusive. The sides in both criminal and civil courts present their version of the “truth.” While in the US the witnesses and the accused are required to take the oath (and may be subject to perjury charges for providing false information), there are provisions protecting the accused from self-incrimination, such as the right to remain silent. The situation is even worse in many European countries, where the protection allows the accused to lie without any consequences (other than lowered trust if their lies are exposed). It is left to humans to sift through these contradictory accounts in an attempt to determine which is closer to the truth. Especially in jury-based systems, the conflict of “differing narrations” is exacerbated by the role of emotions. They determine the degree of belief in the testimonies of the sides, sometimes openly, sometimes less visibly. Taking these difficulties into account one may doubt if AI can be effectively introduced into the core of court proceedings. Such introduction creates an extremely interesting research challenge: can one train an AI to perform on the basis of incomplete (at best) or contradictory information? Can we teach AI to recognize and deal with lies?

### 2.1. Training AI on incomplete, contradicting, or false data

Imagine the problems of training and testing the accuracy of a machine learning system in the situation described above. The inherent discrepancies in the judicial data are case specific. How would one construct a proper training dataset which would reflect the “lies and smokescreens”? How to measure the goal functions and train the AI? And, especially important, how to estimate the validity of decisions of the trained system applied to specific cases? One could additionally question the potential for bias (e.g., preference for “evidence” coming from specific sources, such as the police, or some forms of evidence). The impact of such biases can be significant—as we know from the current, human-based systems. But at least judges and jurors have faces.

At the same time, formulated as a research challenge, the program to optimize AI for environments with incomplete or conflicting/contradictory information is very interesting (Awasthi et al., 2021). Applications such as medical diagnoses are already studied.

There are studies devoted to the prevention of “poisoning” attacks against AI (Gudivada et al., 2017; Jagielski et al., 2018; Khurana et al., 2019). Such attacks are aimed at corrupting the AI/ML processes *via* false or erroneous information. The focus of these works is finding a way to weed out such false or illegitimate data. In the context of the judiciary application, the trick that the AIs will have to learn is how to deal in the landscape where the data is “inherently poisoned.” In a loose analogy, this is similar to the quantum information systems, where no single “true” state can be defined. An interesting research challenge—I am not even sure if solvable (based on the human experience).

### 2.2. Explainability in socio-legal AI

Explainable AI (xAI) attempts to address one of the major weaknesses of the AI approach: the “black box” behavior. In many cases, it is not enough for AI to provide results (data parsing and categorization, decisions), without our understanding of how the process worked. We hope to be able to provide a “narrative”—understandable by humans—explaining how these results were achieved. Not in the language of numbers of hidden layers or feedback parameters, but in more traditional reasoning terms (Longo et al., 2020; Minh et al., 2021).

Opaque decisions are particularly suspicious in the processes determining our lives. We expect that the courts, government representatives, or other institutions will provide a generally understandable rationale behind their actions governing our individual and communal life. Courts and judges are required to describe, sometimes in great detail, how a particular decision was reached. The same criterion applies to tools supporting decision-makers or making decisions autonomously. For example, computer-based recommendation systems (whether used for news on social media, evaluation of loan applications, or decisions concerning parole) may be (justly) distrusted, because we do not know how and why they reach their conclusions. Why does a particular person get an early release from jail, and the other does not? Lack of explanation (often referred to as black-box machinery) immediately leads to accusations of bias, unfairness, and manipulation. An AI decision-making tool is, for many people, the ultimate kangaroo court (Waltl and Vogl, 2018; Deeks, 2019), faceless by definition.

### 2.3. “Facts” can lie, too: Role of AI in information manipulation

There's a reverse side of the potential role of AI in the socio-legal contexts: rather than using it to determine the truth and help to achieve just outcomes, the sides may use their own AI tools to create or manipulate “evidence.” Such possibility has been even discussed in the context of research community (Gu et al., 2022), where we rely on our honesty and trust, and fraud is an exception. In contrast, in other domains (e.g., politics) one observes an already strong role of the creation and dissemination of misinformation or disinformation. Current capacity for “deep fakes” is still (hopefully) limited, but the developments and improvements continue. Photographic, sound, and video evidence can be artfully manipulated, beyond our capacity to distinguish true from false. The potential dangers go beyond politics. AI can also be used to facilitate manipulation of other factual evidence (e.g., electronically stored records in possession of police or persecution), for example in using AI tools attempts to break in into protected (or unprotected) databases and systems to delete or manipulate important records. One can imagine traceless substitution of fingerprint or DNA evidence provided to the court, clearing the suspect in a way that is considered objective and solid—yet is actually false.

The examples provided above refer to AI uses that are very real. Both the manipulations of rich, sensory data (video, audio) and the use of AI in cybersecurity have their legitimate uses. The movie and gaming industries strive to continuously break the current limitations and create new, believable experiences based on computer simulations, and AI tools play an important role there. The need to protect IT infrastructures from malevolent actors becomes even more important in the context of the Internet of Things and the global vulnerability of the supply chains in the Industry 4.0 paradigm. But the same developments may be used by parties with less legitimate intentions. Once, there was a natural barrier of high computational costs-one needed industry-scale infrastructure to achieve certain effects. But this barrier is exponentially diminishing.

So, presumably, there will be a “battle” between the AI-based systems created to manipulate information available for legal processes and those trying to detect such manipulations. The role of the “jury of your peers” might become even more superficial than it is today, with the reliance on experts being complemented/substituted by the reliance on AIs.

At present, it is impossible to say where such process would lead, and if, indeed, we will enjoy improvements in our right for fair legal processes or its deterioration.

## 3. Cui bono? Cui prodest?

A very important question is: who will benefit from introducing AI into various socio-legal processes and situations? Are the benefits justly distributed? Are they generally beneficial for societies and (most) individuals? Or do they serve the interests of selected groups (corporations, governments, wealthy individuals) at the expense of the rest of the society? In the latter case (which I suspect to be true) can we identify who will benefit most and what measures would have to be implemented to protect our rights?

### 3.1. Much ado about bias

The bias found in many AI/ML applications is the most recognized danger (Ntoutsi et al., 2020; Mehrabi et al., 2021). It has also received a lot of public attention and reaction. The most cited examples are police profiling, AI uses in judiciary decisions, or the AI-related biases in credit application processing, property valuation, medical services and even facial recognition. These biases turn out often to correlate with race or ethnicity stereotypes—which adds importance and gravity to the discovered biases.

The examples cited above have often immediate, life-changing consequences. But all profiling has consequences. As we increasingly move through the digital world “guided” by prompts, suggestions, and ads curated by unknown algorithms, we might be unaware that the vision of the world we see is skewed. That our own decisions are not wholly “our own.” AI/ML sifts through our digital trails, optimizing the world we see with respect to goals set out by advertisers or politicians. Often this “guidance” is not even recognized by us, or—worse—we mistake the matrix of the algorithmic bubble for reality and universal truth. It takes significant, conscious effort to seek beyond what is prepared for us.

A similar (and connected) situation was realized some time ago with respect to the personal information handling. The regulators recognized that many people do not understand the potential for abuses of the data identifying them. At the same time, the needs of companies and institutions to effectively provide the services (a.k.a. “legitimate interests”) had to be considered. The resulting GDPR regulations, such as the one present in the European Union, solves some problems, protecting certain types of information from processing or making the fact of such processing known. The limits on the gathered data, legitimate uses, the right to be “forgotten” provide at least some rights to the individuals. Unfortunately, these protections do not work everywhere or vary from country to country. Multinational companies operate in geographies with widely differing legal protections of personal data. Second, what about the people who automatically click the “agree” buttons? Those who do not understand the consequences or simply do not care? Third, even in countries where strict GDPR rules are in place, how many people are actually remembering who has their data and who actively invoke their rights, like the right to be forgotten? Who check how the data is processed or used? The same questions, regarding the effectiveness of top-down legal protection have to be asked in the cases of AI/ML enhanced profiling.

These consequences of the asymmetry between individuals and institutions are not limited to the careless and the vulnerable. Of course, they bear the immediate brunt of the results of profiling. But an important question arises: what may be the general impact of training of AIs used in profiling on data from this sub-population, rather than a representative one? Imagine policy makers who use for their decisions analyses based on the most active social media/Web users, with clearly articulated political views. Those who share are often the ones with extreme views, attacking their opponents and showing high levels of partisanship. The moderates, the cautious, the undecideds are not so open to having their voices heard (and analyzed)—often they form a “silent majority.” Thus, both the politicians and the AI systems analyzing social preferences may work on generally biased data (e.g., showing more polarization than is present in reality or preference for populist solutions). Based on the social preferences deduced from such imperfect data processing, decision makers would create policies that cater to wrong expectations (e.g., exacerbating the polarization, or shifting policies to populism).

### 3.2. The rich get richer or power to the people?

Democratic societies place great value on equality under the law—including the right to legal counsel. One can ask if AI tools change this equality? With enough money and efficient tools (including AI/ML) a large company can gather far more information about their opponent, competitor, partner, and employee—creating an advantage in legal processes, business negotiations, complaint handling etc. In the US: “My AI is better at finding relevant precedents than yours.” Of course, this lack of balance is already present in the current system. But would the use of AI tools to sift through mountains of subpoenaed evidence in civil cases create another level of inequality?

Or, perhaps, the ubiquity of the tools would actually provide a better landscape for everyone when used to evaluate information acquired *via* the Freedom of Information Act or its equivalents. Of course, the socio-legal landscape is more than just court proceedings. In the knowledge economy, access to data and the capacity to process it effectively and obtain important insights from it provides a significant competitive advantage.

Today, the balance of power seems to favor the global corporations and governments: they have the data, the money to create tools and to pay developers, and—very importantly—they already have definite goals in mind. Sometimes “good goals”: better products, better healthcare, national security and the fight against terrorism. Sometimes bad ones: unscrupulous profit raking or controlling the society. Can this imbalance be corrected? It depends, in part, on what we (scientists) would concentrate on developing.

### 3.3. Power corrupts

One can not forget the role of the state in the socio-legal landscape. The asymmetry between the power of the government and its agencies and the citizens or their associations is well recognized. Most of the legal systems in democratic societies explicitly include measures to protect us from the consequences of this asymmetry. These rules impose limitations on the capacities of the state, partially balancing the unequal division of power and resources. But are these solutions (tracing back to medieval times) adequate in modern societies?

Ever since the beginning of the growth of electronically networked society, some people wanted the capacity to “get off the grid,” to avoid the potential monitoring and control of their activities by “the authorities” (whatever they might be). With more and more of our life moving to the virtual (or even simply connected) world, the amounts of data available become truly fearsome. Probably in all countries, the governments, or at least some specialized agencies, have the right to access these datasets. For the purposes of providing social services, health services, safety—or national security and law enforcement. In democratic societies, we can at least hope for some accountability or a minimal level of transparency or court control. But what about more autocratic countries, like Russia or China? There are serious concerns about the abuses and misuses of AI in these countries (Ahmed et al., 2019; Polyakova and Meserole, 2019; Zeng, 2020; Shi, 2022).

### 3.4. The dark side: AI for crime?

One of the often forgotten aspects of scientific progress is the potential dual-use of the research results. In some disciplines, this is recognized explicitly (nuclear physics, chemistry, and engineering; biology—especially of contagious diseases and gain-of-function studies; missile technologies, etc.). Some aspects of AI are also classified as dual-use, and partially regulated. The prospect of AI “arms race” and cyberpace wars is well recognized—but calls such as Taddeo and Floridi (2018) remain just this—calls for action. But the rapid growth of the available tools, ever-cheaper computing power, and data storage make the top-down restrictions and monitoring largely impractical. The costs go down and data access easier, so the natural “non-proliferation” cost/technology barrier is lowered. The tools, once available only to the “large” players (states, large corporations), become feasible for anyone smart enough to find a profitable use. And crime falls into this category. Fraud, identity theft, and blackmail would clearly benefit from the advanced capacities offered by AI tools. Other nefarious applications are certainly possible—for example creation of new drugs. Unfortunately, the topic is only relatively lightly studied (King et al., 2020).

## 4. Conclusions: Unforseen consequences

The preceding sections discussed selected ethical problems associated with the development of AI tools used in various socio-legal contexts. Some of them are already well publicized, such as biases present in ML-based selection/recommendation algorithms (often reflecting preexisting biases in their human equivalents) or dangers of using AI face recognition on a massive scale. Other are less recognized, but this lack of recognition might mean that we have not (yet) considered the consequences deeply enough.

We (the research community) are trapped in the race for results, the cycle of grant proposal preparations, our individual careers, and institutional priorities. How often are we ready to stop and think about the unforeseen or undesirable “impact” of our research? This question is not limited to AI/ML studies and the efforts to develop better algorithms and applications. Physicists designing new materials, biologists using ever-better tools for genetic manipulation, linguists studying patterns separating human and machine-generated texts—we are all passionate about our research. In the grant proposals, we proudly claim (as required!) the positive impact of our studies: new, ecologically friendly materials, potential cure of genetic diseases, fight with spam and hate-spewing bots…In many cases, the “ethics clauses” are treated perfunctorily, lest the funding authorities get scared and refuse to fund if we point out too serious problems in our applications. New materials for tank armor or better explosives? Genetic manipulation? Building better spam bots? Not us, surely. The current research landscape reverses Galileo's openness: he has promoted the military potential of the telescope in parallel with the study of stars.

This disregard for ethical considerations is, in my experience, even more characteristic of scientists who (like me) come from the STEM fields. Armed with our tools (whether they are differential equations, computer simulations or AI/ML) we think of expanding the “the rigor” of STEM to humanities and social sciences. The “unwanted consequences” and human angle are often marginalized because they are outside our previous experiences. Atoms do not complain when exposed to high pressure and temperature. Nor do galaxies object to being classified by an AI/ML algorithm. Nobody finds an issue with massive “uninteresting” data being rejected in CERN LHC experiments^3^. But when we would move to studies of human activities the situation changes. Not technically, but ethically. We know that dropping the data is dangerous. Not just research-wise, but also when scientific results obtained on skewed or biased data are used to support general policies, for example in healthcare. Similar ethical doubts are connected with practices: experimenting with people, stereotyping them *via* some algorithms or selecting data to focus on “important” groups at the expense of others are no-go territory. Or are they? We are smart, and this includes being good at hiding the questionable potential outcomes of our research and promoting the desired (or popular) ones. And the grant application reviewers are often in the same boat, so they may turn a blind eye for the sake of “excellent research.”

Even when university Ethics Boards do their job, and limit our “academic freedom” for the right reasons, even when we actually listen to the warnings coming from concerned NGOs, what is once discovered can not be “covered back.” Governments, companies, and crime syndicates could use our tools just as Galileo's telescope found its way to royal navies, merchant ships and pirates' hands. In his case, the instrument was at least equally defensive as offensive: it allowed not only to seek the enemy, but also to steer away from the danger with better warning. And here may be the source for the closing thought: maybe we should focus on these applications of AI in the socio-legal domain that—by design—favor protecting our rights and freedoms.

In addition to concentrating our research efforts on socially beneficial applications (as contrasted to purely technical advances) there are legal paths that can be taken. The recently announced BLOOM Large Language Model created by researchers from over 70 countries within the BigScience project has considered (Danish Contractor et al., 2022) and developed a dedicated *Responsible AI License*^4^. The license expressly forbids the use of the model or any derivatives in several domains, including socio-legal contexts. In particular, it forbids the use of the tools “*For fully automated decision making that adversely impacts an individual's legal rights or otherwise creates or modifies a binding, enforceable obligation; For any use intended to or which has the effect of discriminating against or harming individuals or groups based on online or offline social behavior or known or predicted personal or personality characteristics; […]; For any use intended to or which has the effect of discriminating against individuals or groups based on legally protected characteristics or categories; […]; To generate or disseminate information for the purpose to be used for administration of justice, law enforcement, immigration or asylum processes, such as predicting an individual will commit fraud/crime commitment (e.g., by text profiling, drawing causal relationships between assertions made in documents, indiscriminate and arbitrarily-targeted use)*.” This form of protection of our rights by incorporating use conditions into licenses is a significant step forward from the previous attempts to regulate the use of AI tools, based on ethical *appeals*, like the Montréal Declaration. It moves the restrictions onto enforceable, legal grounds. Should this approach gain universal popularity, the AI tools could regain the balance between societal responsibility and development.

## Author contributions

PS has conceived and written the present paper.

## Conflict of interest

The author declares that the research was conducted in the absence of any commercial or financial relationships that could be construed as a potential conflict of interest.

## Publisher's note

All claims expressed in this article are solely those of the authors and do not necessarily represent those of their affiliated organizations, or those of the publisher, the editors and the reviewers. Any product that may be evaluated in this article, or claim that may be made by its manufacturer, is not guaranteed or endorsed by the publisher.

## Footnotes

^1^Note that categories of good/evil and intended/unintended are not identical: it is possible to imagine uses that are intended and evil. One has to evaluate both the *intentions* and the *outcomes* of the AI implementation.

^2^A recent example is the comprehensive data harvesting policy of TikTok (https://www.tiktok.com/legal/new-privacy-policy?lang=en). The scope of the data collected by TikTok is so great that it caught the attention of a member of the US FCC. It comprises—among other categories—of search and browsing history, photographs (including facial ID), voice samples, location data etc. While the reason of the inquiry is the potential access to the data from China, rather than the US, the scope of the data gathered is very broad. When we couple this information with the statistics that 27% of TikTok users are below 17 years old group (https://www.oberlo.com/statistics/tiktok-age-demographics), the implications are rather serious.

^3^ https://www.lhc-closer.es/taking_a_closer_look_at_lhc/0.lhc_data_analysis

^4^ https://huggingface.co/spaces/bigscience/license
